# Recognition, Diagnosis, and Treatment of *Clostridioides difficile* Enterocolitis Presenting Without Diarrhea: A Literature Review

**DOI:** 10.3390/pathogens14020181

**Published:** 2025-02-12

**Authors:** Ezgi Yılmaz, Duygu Işıl Gencer, Mustafa Salih Akın, Meyha Şahin, Bahadır Ceylan, Ali Mert

**Affiliations:** 1 Department of Infectious Diseases and Clinical Microbiology, Faculty of Medicine, Istanbul Medipol University, 34815 Istanbul, Turkey; meyha.sahin@medipol.edu.tr (M.Ş.); bceylan@medipol.edu.tr (B.C.); alimert@medipol.edu.tr (A.M.); 2 Department of Obstetrics and Gynecology, Faculty of Medicine, Istanbul Medipol University, 34815 Istanbul, Turkey; duygu.isil@medipol.edu.tr; 3 Department of Gastroenterology, Faculty of Medicine, Istanbul Medipol University, 34815 Istanbul, Turkey; msakin@medipol.edu.tr

**Keywords:** CDI, ileus, *difficile* enterocolitis, CDI without diarrhea

## Abstract

Diarrhea, as the well-known clinical feature of *Clostridioides difficile* infection (CDI), may be absent at the initial presentation, leading to delays in diagnosis. The delay is due to both underrecognition of such presentations and the dependence of CDI diagnosis on stool samples. This review was conducted to evaluate the literature for CDI cases presenting without diarrhea, raise awareness about the possibility of CDI in the differential diagnosis regardless of diarrhea, and assemble relevant data to harmonize clinical approaches. The PubMED Medline database was used to conduct this literature review, focusing on reported CDI cases presenting without diarrhea. After exclusions, 22 articles were included for analysis, providing data for 48 cases. This paper will present the selected clinical data of these 48 patients and follow a real-life case with a clinical course of CDI including presentation, diagnosis, management, and outcomes. The excessive mortality and bowel resection rates of CDI patients presenting without diarrhea were the notable findings. Poor prognosis was possibly inflated by delayed diagnoses in an unfamiliar setting, emphasizing the importance of a high index of suspicion to allow early recognition of CDIs in the appropriate clinical context despite the absence of diarrhea.

## 1. Introduction

*Clostridioides difficile* infection (CDI) is defined by the presence of symptoms (usually diarrhea) and either a stool test positive for *C. difficile* toxins or detection of toxigenic *C. difficile*, or colonoscopic or histopathologic findings revealing pseudomembranous colitis in the Clinical Practice Guideline for CDI, by the Infectious Diseases Society of America (IDSA) [1]. Demonstration of the toxin or isolation of the *C. difficile* toxigenic strain from stool samples is diagnostic of CDI in appropriate clinical settings. Colonoscopic examination and biopsy can also provide evidence for the presence of CDI by visualizing the typical features described as pseudomembranous colitis [2]. CDI cases may range in clinical severity from asymptomatic carriage to profuse diarrhea, and occasionally to full-blown colitis complicated by toxic megacolon [3]. Rarely presented features of CDI include constipation, abdominal distension, isolated abdominal pain, or postoperative prolonged ileus, which have been sporadically reported in individual case descriptions or small case series. Reports suggesting an increasing CDI incidence in settings previously considered as low risk for CDIs (non-nosocomial, obstetric, etc.) have also been concerning. CDI diagnosis can be easily overlooked in practice with rare presentations due to their underrecognition and underrepresentation resulting in delayed or missed diagnosis, which may have serious consequences. To make matters worse, many laboratories only test stool for CDI in the presence of diarrhea, further reducing the probability of timely diagnosis when CDI presents with less familiar features [2]. Appreciating the current limitations in our understanding of CDI cases with rare presentations, the data from a laborious systematic literature review focusing on the recognition, diagnosis, and management of CDI presenting without diarrhea are displayed in this paper. The clinical course of a real-life case is described and discussed in relation to the literature. The aim was to raise awareness of rare CDI presentations by assembling relevant data and elaborating on ways to harmonize clinical approaches.

## 2. Material and Methods

**Figure 1:**
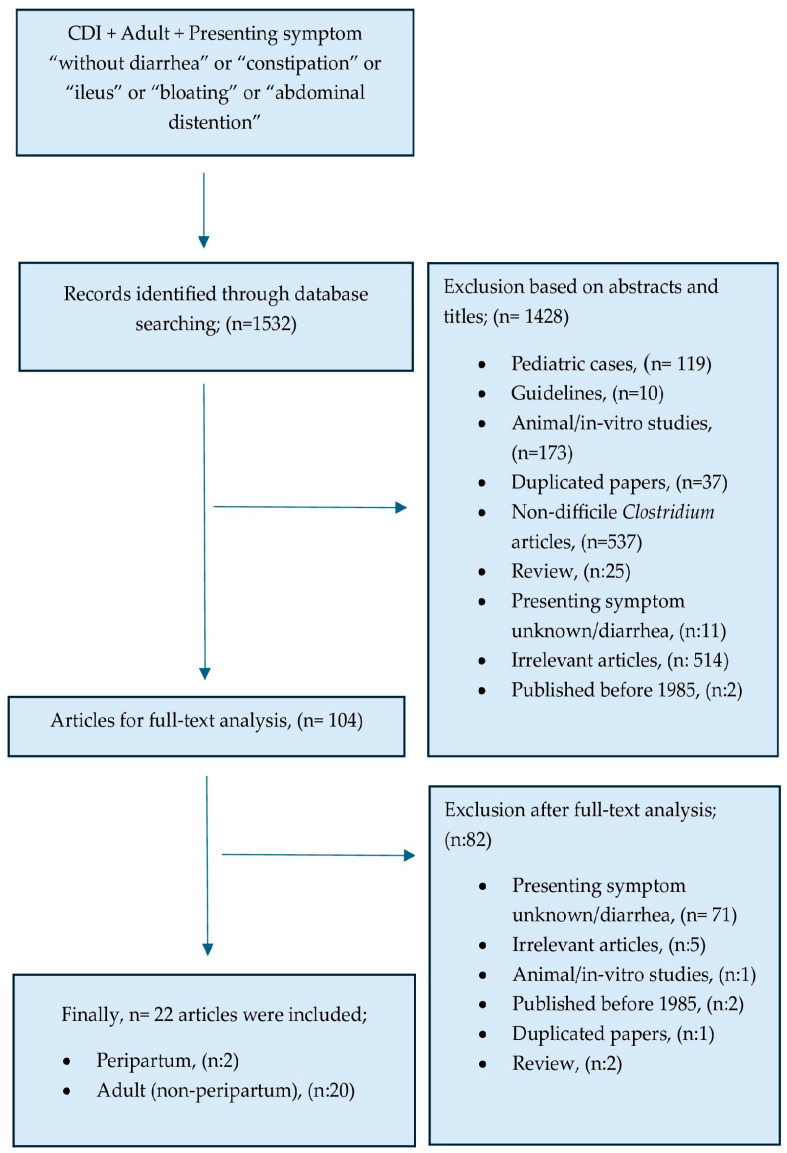
Flowchart for literature review.

We conducted a literature review using two different keyword sets to assemble all relevant data from the MEDLINE PubMED database (Figure 1). The first keyword set focused on CDIs presenting without diarrhea in adult patients (age > 18) using the combination “(((*difficile*) OR (pseudomembranous colitis) OR (*clostiridium difficile*)) AND ((ileus) OR (constipation) OR (obstipation) OR (bloating) OR (abdominal distention)))”. For the second search focusing on CDI in the obstetric setting, we used the following keyword combination,“(((pregnancy) OR (peripartum) OR (postpartum)) AND ((*clostridium*) OR (*difficile*) OR (pseudomembranous colitis) OR (pseudomembranous enterocolitis))). The search was conducted on the 3 of February 2025. Initial screening involved reviewing titles and abstracts to select relevant papers among all articles reached with the searched keyword combinations. Full texts were also reviewed during screening in cases where abstracts did not provide information regarding symptoms at presentation in the screening phase, and reported cases presenting with any severity of diarrhea were excluded only after full-text evaluation. Selected articles after screening were carefully analyzed and relevant data extracted. Papers not reporting novel information or providing case data regarding CDIs presenting without diarrhea were categorized as irrelevant and excluded from the analysis.

The case data described in the manuscript were retrieved retrospectively from institutional records. The patient signed an informed consent for the anonymous use of her clinical data for research purposes. The study was conducted and the manuscript prepared in accordance with the Declaration of Helsinki, strictly adhering to good clinical practice.

## 3. Results

### 3.1. Literature Overview

The two keyword combinations yielded a total number of 1532 articles. The flowchart of the screening, exclusion, and selection of these papers is shown in Figure 1. Full-text review was performed for 104 articles, and 22 articles were found relevant for the purposes of this review and included in the analysis. These 22 articles provided information about 48 patients who were diagnosed with and managed for CDI after presentation without diarrhea (Table 1 and Table 2). Adequate information on prior antibiotics, treatment, and diagnostic procedures was reported for 35 of these cases, whereas outcome data were available for 43 cases. Overall, 13 of these 43 cases (not including the clinical case discussed in this manuscript) succumbed to complications during CDI and surgery was needed in 13 cases, with most resulting in extensive bowel resection. Data regarding fever and leukocyte count at presentation were missing for about half of the patients (56% and 42%, respectively). Imaging modalities and data regarding inciting antibiotics were not available for 43.8% and 34% of the patients, respectively. Among the patients, about one-third had no medical treatment data available. Outcome data were missing for about 11% of them.

### 3.2. Clinical Case

#### 3.2.1. Background and Presenting Symptoms

A 36-year-old, previously healthy, multiparous (G3/P2) pregnant woman underwent cesarean section (C/S) in the 36th week of pregnancy and was discharged from hospital two days after C/S. One week after the delivery, the patient presented to the emergency clinic with severe abdominal pain, lack of gas, stool discharge since C/S, and intractable vomiting.

#### 3.2.2. Initial Evaluation and Recent History

**Figure 2:**
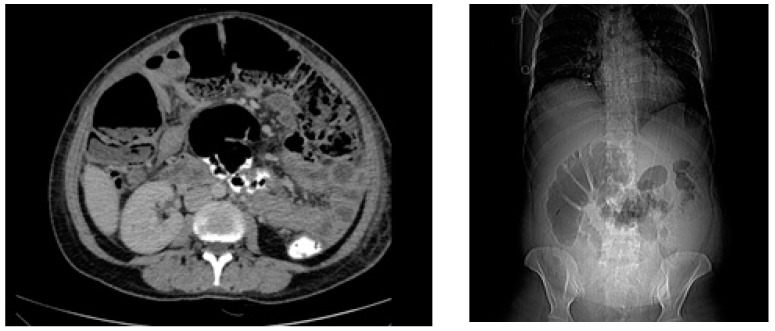
Abdominal CT at presentation indicating colonic dilatation with colonic wall thickening was consistent with ileus without obstructing mass or collection.

The patient appeared critically ill, and she was febrile (38.4 °C); her blood pressure was 100/55 mmHg, and she was tachycardic (118 bpm). The physical examination was significant for diffuse pain on all four quadrants to palpation, hypoactive bowel sounds, and severe abdominal distension without rebound tenderness consistent with acute abdomen with prolonged postoperative ileus. There was no discharge from the site of incision. At presentation, laboratory tests were remarkable for anemia (Hgb = 7.7 g/dL) marked elevation in acute-phase reactants (CRP = 425 mg/L; procalcitonin = 0.33 ng/mL), and white blood cells with neutrophilic predominance (leukocytes: 14,100 cells/mm^3^; neutrophils: 85.4%). Abdominal CT was consistent with ileus without an obstructing mass or postoperative collection (Figure 2). Her recent obstetric history was unremarkable other than the administration of oral third-generation cephalosporin (cefdinir 600 mg q24h) for asymptomatic bacteriuria for five days on the 34th week of pregnancy. Postpartum, she had also used two days of cefdinir per oral as postoperative prophylaxis. After ruling out mechanical obstruction and surgical site infections, fulminant CDI was considered in the differential diagnosis based on the severity of the clinical picture, recent operation, hospitalization, and antibiotic use.

#### 3.2.3. Diagnosis of CDI

After 24 h of admission, clinical status was not improved despite empirical wide-spectrum antibiotherapy (piperacillin–tazobactam) and supportive care. Abdominal discomfort was not relieved, and an urgent colonoscopy was performed on day 2, which demonstrated pseudomembranous patchy involvement and black ground ulcerative lesions in the cecum and ascending colonic mucosa. Highly suggestive features for CDI were observed, so fecal samples were collected via a rectally inserted tube and found positive for *Clostridioides difficile* toxin A/B by immunochromatographic methods along with positivity for glutamate dehydrogenase (Standard F *C. difficile* Toxin A/B FIA, SD Biosensor, India). Pathological examination of the colonic biopsy was consistent with pseudomembranous colitis by *C. difficile*.

#### 3.2.4. Management of CDI

The patient was given vancomycin per oral(125 mg qid) plus metronidazole (500 mg tid) parenterally immediately after colonoscopy. Because of clinical instability, piperacillin–tazobactam was also continued. Two days after the onset of antibiotherapy, watery, non-bloody diarrhea of moderate severity was observed. On day 3, abdominal CT was repeated due to recurring abdominal pain, demonstrating a new fluid collection near the surgical incision. Specimens cultured from the drainage of this collection did not result in bacterial growth and the collection disappeared over the following days. On day 7, the clinical status was stabilized, and the diarrhea had resolved. The antibiotic combination was continued for 14 days, and the patient was discharged from the hospital in excellent health, maintaining her good condition in her outpatient visit after one year without recurrence.

### 3.3. Literature Review: CDI in the Obstetric Setting

The incidence of CDI in peripartum women has increased in the last 20 years [26,27]. Several risk factors for CDI have been described in the peripartum period such as cesarean section and antibiotherapy for asymptomatic bacteriuria, which is frequently administered in the last trimester. Prior antibiotherapy and cesarean delivery are recognized as predisposing factors for peripartum CDI, due to prolonged hospital stays and more frequent antibiotic use with C/S [27,28,29,30]. The risk of CDI increased with increasing numbers of antibiotics, cumulative doses, and the duration of antibiotherapy [29]. The most common antibiotic related to CDI was also cephalosporins in obstetric settings; all three patients including the case presented in this paper had used cephalosporin before the onset of CDI. Saha et al. stated that peripartum women diagnosed with CDI had a median of 2 days (range 1–6) of recent antibiotic use before the onset of symptoms [4]. The authors also emphasized that most of the CDI cases developed on days 4–9 of antibiotherapy, with the range extending up to 8 weeks after the end of antibiotic exposure.

### 3.4. Literature Review: CDI Presenting Without Diarrhea

CDI presenting without diarrhea is not uncommon in clinical practice. Experienced physicians commonly consider the possibility of CDI in the right clinical settings despite the absence of diarrhea [10,17,22]. However, these settings are underrepresented in the literature, and an earlier paper describing five cystic fibrosis (CF) patients with CDI argued that constipation during CDI could be attributed to CF [9]. Reported cases over time established that diarrhea was not an essential feature to suspect an ongoing CDI [10,20,22,24]. Between 1985 and 2024, forty-eight patients diagnosed with CDI in the absence of diarrhea were reported (Table 1 and Table 2). The median age was 59 years (range 21–82), and 58% were male. Previous medical history was available for 85% of the cases. Among them, eleven (26.8%) had hematologic or solid organ malignancy, six had cystic fibrosis, and two of them had a solid organ transplantation history. The predominant symptoms were constipation and/or abdominal distension in 38 of these 48 cases (79.1%). Body temperature was ≥38 °C in 61.9% (13/21) of patients at presentation, and 92.8% (26/28) had elevated white blood cell counts, with most of these being above 15,000 cells/microL. Previous antimicrobial use was documented in all but one of the reported patients (Table 1). Beta-lactams (two-thirds cephalosporins) were the most commonly prescribed antibiotics (*n*:19), followed by quinolones (*n*:8), cotrimoxazole (*n*:5), and clindamycin (*n*:4). Previous vancomycin use alone or with other antibiotics was also reported for eight cases. Data on abdominal radiography and/or computed tomography were available in 27 of the patients, and large bowel dilatation was the most consistent finding; colonic wall thickening suggestive of colitis was observed in 11 of these cases (Table 1).

### 3.5. Literature Review: Diagnosis of CDI in the Absence of Defecation

Rampling et al. and Triadafilopoulos et al. were among the earliest researchers to report CDI presenting with ileus in 1985 and 1991, respectively [4,5]. These papers document eight cases of CDI without diarrhea, among which six were diagnosed via urgent colonoscopy (Table 2). In 2001, Sheikh et al. reported a case of pseudomembranous colitis without diarrhea presenting clinically as acute intestinal pseudo-obstruction, who did not respond to conservative management and then underwent colonoscopic biopsy, which returned positive for *C. difficile* toxins despite stool samples being negative [11]. These earlier reports were pivotal in establishing that CDI could present without diarrhea and raising awareness of this phenomenon. These papers also emphasized the potential of stool testing in evaluating ileus. Despite the absence of diarrhea at the presentation, stool samples were obtained through various interventions in 43.7% (21/48) of cases reported in the literature (through digital disimpaction, *n*: 5; after lactulose laxative use, *n*: 3; via rectal tube, *n*: 3; after enema, *n*: 2; defecation after suppository use, *n*: 2; defecation after colonoscopic decompression, *n*: 2; sampling from ileostomy post-surgically, *n*: 2; stool sampling during colonoscopy, *n*: 2) (Table 2) [10,11,12,13,14,18,19,20,22,23,24,25]. The method was not specified for 12 patients from whom stool samples were obtained. Tests on stool samples included latex particle agglutination (*n*: 6), cytotoxicity assays (*n*: 3), *C. difficile* toxin assays (*n*: 12), *C. difficile* toxin PCR testing (*n*: 8), and stool cultures (*n*: 4). Testing for stool samples attained through various interventions was pivotal in reaching a CDI diagnosis and administering potentially life-saving treatments in these 21 cases. Urgent colonoscopy (or proctoscopy/sigmoidoscopy) was performed in 14 cases, demonstrating pseudomembranous colitis in 85.7% (12/14) of these cases, which established or confirmed CDI diagnosis (Table 2). No complication associated with colonoscopy was reported among these 14 cases. The in-hospital mortality was 1 in 14 among those undergoing emergency colonoscopy, while 12 of the remaining 34 cases succumbed to CDI. Although compelling, this comparison was statistically insignificant (*Fischer’s exact test p* = 0.07). Surgical exploration for diagnostic purposes was performed in five cases (Table 2). Two cases of CDIs presenting without diarrhea in the obstetric setting were also previously reported (Table 2) [15,25]. Both patients were exposed to beta-lactams, presented with ileus, and diagnosed with CDI by testing stool samples for *C. difficile* toxin.

### 3.6. Literature Review: Management of CDI Presenting Without Diarrhea

Information regarding antibiotherapy for CDI was available for 27 (56%) of the 48 patients in the literature (Table 2). Metronidazole (monotherapy or combination) was the most common antibiotic administered to patients with CDI who presented without diarrhea (88.8%), mostly by the intravenous route. Vancomycin was administered either orally or colloquially in 53.8% of patients, and 26.5% of patients received a combination of vancomycin and metronidazole (Table 2). Five of thirteen (38.4%) patients receiving metronidazole monotherapy died, while the outcomes of eight other patients receiving metronidazole therapy could not be clarified; four of these may have also succumbed to the disease. Twelve of fourteen patients (84.6%) receiving vancomycin (either alone or in combination with metronidazole) survived the event (*Fischer’s exact test p* = 0.36). Colonic resection was reported in 13 of these 48 patients (27.0%); however, surgical outcomes were not specified in detail consistently. Bowel resection was needed in six among twelve patients treated with metronidazole alone and two among ten patients receiving combination antibiotics. We did not detect any variable with statistical significance for mortality between antibiotics, surgery, and colonoscopic evaluation, mainly due to the small sample size. Thirteen of the forty-three CDI patients (30.2%) who presented without diarrhea succumbed to the disease and the outcomes for the remaining five cases were not reported. Among the 30 survivors, 7 (23.3%) had to undergo extensive bowel resection while 1 patient suffered persistent kidney failure starting on renal replacement therapy after the CDI episode (Table 2). Two of the three cases of CDIs presenting without diarrhea in the obstetric setting, including the case reported in this paper, received a combination of metronidazole and vancomycin and fully recovered. The other obstetric case was delayed in diagnosis due to confounding factors, received IV metronidazole monotherapy, and had to undergo extensive bowel resection.

## 4. Discussion

Deviation from the familiar patterns of presentation or evolution of diseases is confusing for clinicians. Delays in timely diagnosis as well as potentially life- or organ-saving treatments are common in such circumstances. Patients presenting with constipation or ileus are less likely to be considered and tested for CDI due to the underrecognition of this feature, and the difficulties and risks of attaining stool samples in such a setting. On the other hand, physicians should be aware of the concept of colonization with *Clostridioides difficile* in patients without abdominal symptoms (and make sure that incidental findings on stool tests correspond to relevant clinical manifestations of CDIs as outlined in the literature overview (Table 1) to avoid overdiagnosis [3]

CDI in general is a severe disease associated with high morbidity and mortality. The 30-day mortality ranged between 5 and 34% in the literature [31,32,33,34]. This literature review focused on CDI presenting without diarrhea documented a mortality rate at the higher end of this spectrum at 30.2% (13/43), confirming that such a presentation can be indicative of a severe form of CDI. Considering the difficulties in recognition and diagnosis of CDIs with such presentation as discussed above, an earlier report () suggested that many cases may have gone undiagnosed, and the actual incidence and mortality rate may be even higher [22]

Data assembled through this literature review highlighted some key features. First of all, most of the diagnoses reported in the literature were products of a high index of suspicion for CDI. This was mainly noticeable through efforts to obtain stool samples in various ways to test for CDI in 21 cases. It was hardly possible for most of these 21 patients to be diagnosed without careful recognition of the possibility of CDI. Another noteworthy observation was the performance of urgent colonoscopy and the accurate recognition of the colonoscopic appearance, which may have contributed to timely management since patients undergoing urgent colonoscopy mostly survived without a complication (mortality undergoing colonoscopy/sigmoidoscopy: 1/14 vs. 12/34; *p* = 0.07).

The other key feature was the high frequency of the presence of poorly prognostic variables for the commonly used Zar score [35] (leukocytosis, fever, older age) among cases of CDI presenting without diarrhea. Potentially high Zar scores coupled with the high mortality and resection rates documented in this study confirm the gravity of this setting. Observing that only 27 of the 48 patients survived without bowel resection, we can confirm the unmet needs in CDIs presenting without diarrhea.

There are currently insufficient data to generate unanimous recommendations for the diagnosis and management approaches specific to CDIs presenting with ileus, considering the limitations of the available literature presented in this paper. The key limitations of the literature include but are not limited to the low number of reported cases, unavailability of data for leukocyte counts, fever, imaging details, antibiotic durations, and outcomes in a proportion of patients, as well as heterogeneous practices across reporting centers. The high mortality and morbidity rates reported with this pooled analysis are rough estimates at best, due to the influence of both publication bias, which may contribute to the overestimation of morbidity, and the unknown outcomes of possible cases that were undiagnosed. The assembled cases make it clear that CDI can present with ileus and its timely diagnosis requires a high index of suspicion, whereas its true incidence among patients presenting with non-mechanical ileus is yet to be explored. Appreciating the possible complications of high-volume enemas or colonoscopy in the presence of ileus, a cautious approach to attaining stool samples by prioritizing the minimally invasive and safest methods is called for. To the best extent of our understanding, CDI should be considered more strongly in the differential diagnosis of ileus with recent antibiotic exposures, hospitalization, absence of mechanical obstruction in non-invasive imaging, and the absence of other common causes such as electrolyte anomalies or opioid use. The stronger the consideration, the more invasive methods such as higher-volume enemas or colonoscopies may be justified. A multi-disciplinary approach with the involvement of gastroenterology and general surgery would ensure patient safety. Regarding treatment, a tendency towards better outcomes (numerically fewer deaths and bowel resections) with vancomycin is in line with general principles in managing CDIs, and preference of vancomycin should be strongly considered (for fulminant CDI, *strong recommendation, moderate quality of evidence*) [1]. Although none of the 48 patients reviewed here received fidaxomicin, data from trials are encouraging (*conditional recommendation, moderate quality of evidence*) for patients with an initial episode of CDI, with costs and availability remaining as obstacles [36]. The addition of intravenous metronidazole, especially when ileus is documented, and considerations for surgical interventions need to be individualized as recommended for all CDI patients [1]. Fecal microbiota transplantation (FMT), another important treatment modality for recurring CDIs, is yet to be explored in the setting when CDI presents without diarrhea [36].

## 5. Conclusions

CDI presenting without diarrhea can be associated with high mortality and morbidity rates, potentially exceeding those of typical CDI cases. Delay in diagnosis due to unfamiliar presentation is a likely contributor to this poor prognosis. We encourage the consideration of CDI in the differential diagnosis of ileus or megacolon in the appropriate setting of previous antibiotic use or hospitalization, especially if imaging is not suggestive of mechanical obstruction. Increased awareness and prompt actions to reach diagnosis early in the course by attaining stool samples with the safest methods possible may allow timely CDI diagnosis and reverse the unfavorable prognosis. Vancomycin treatment, as recommended for all CDI cases, may be associated with improved outcomes.

## 6. Future Directions

We consider that the true incidence and mortality of CDIs presenting without diarrhea may be higher than that represented in the literature due to diagnostic challenges and underrecognition among clinicians as discussed throughout the manuscript. It may be worthwhile to formulate standardized reporting protocols for atypical CDI presentations as prospective registries incorporating data for presenting symptoms, vital signs, comorbidities, leukocyte counts, details of prior antibiotic use, recent hospitalizations, surgery, and diagnostic procedures, along with doses and duration of CDI-directed therapies and surgical interventions. Carefully collected data in the form of such standardized protocols will not only raise awareness but also allow us to better understand this challenging condition.

## Figures and Tables

**Table 1:** Background features and initial evaluation of 48 patients diagnosed with CDI in the absence of diarrhea.

Case No [Ref]	Year	Age/Sex	Comorbidity	Signs and Symptoms	Fever (°C)	Leukocytes (Cells/mm^3^)	Imaging Findings
[4]	1985	69/F	ALL	Abdominal distension, peritonitis	-	Neutropenia	-
2. [4]	1985	45/F	AML	Abdominal distension	Yes	27,000	Ascending colon dilatation/CT
3. [5]	1991	63/M	NHL	Constipation	38.3	-	USG
4. [5]	1991	65/M	Metastatic prostate ca	Abdominal distension	39	12,100	Colonic wall dilatation/X-ray
5. [5]	1991	66/M	COPD, lung ca	Abdominal distension	No	25,800	X-ray
6. [5]	1991	42/M	Peptic ulcer, alcoholism	Abdominal distension, acute renal injury	39.2	17,100	X-ray
7. [5]	1991	76/M	Heart failure, stroke, renal failure	Abdominal distension	No	8900	X-ray
8. [5]	1991	68/M	COPD	Abdominal distension, obstipation	38	18,600	X-ray
9. [6] *	1992	84/F	-	Abdominal pain, obstipation	-	-	X-ray
10. [6]	1992	-	-	Abdominal pain, obstipation	-	-	-
11. [6]	1992	-	-	Abdominal pain, obstipation	-	-	-
12. [6]	1992	-	-	Abdominal pain, obstipation	-	-	-
13. [6]	1992	-	-	Abdominal pain, obstipation	-	-	-
14. [7]	1998	-	AF Renal failure	Constipation	-	-	-
15. [8]	1999	44/M	Renal failure	Constipation	-	Elevated	-
16. [9]	1999	21/M	Cystic fibrosis	Constipation	39.5	27,000	Pancolonic bowel wall thickening/CT
17. [9]	1999	41/M	Cystic fibrosis	Constipation	39.5	27,000	Pancolonic bowel wall thickening/CT
18. [9]	1999	22/F	Cystic fibrosis	Constipation	39.5	23,000	Pancolonic bowel wall thickening/CT
19. [10]	2000	70/F	COPD, AF, HT, heart failure	None	-	14,300	Cecum dilatated/CT
20. [11]	2001	67/F	HT, DM, heart failure, COPD, renal failure	Abdominal distension, obstipation	No	22,900	Colitis/ray + CT
21. [12]	2004	44/M	Polyneuropathy	Constipation, vomiting	39	15,000	Colonic wall dilatation/ray + CT
22. [13]	2005	60/M	Gastric lymphoma	Abdominal pain	-	Elevated	Colonic wall dilatation/CT
23. [14]	2007	28/M	Cystic fibrosis, lung transplantation	Abdominal distension	-	-	Thickened large bowel suggestive of colitis/CT
24. [14]	2007	22/M	Cystic fibrosis, lung transplantation, acute rejection	Abdominal pain, constipation	-	Elevated	Colonic dilatation suggestive of colitis/X-ray + CT
25. [15]	2007	-	Postpartum	Abdominal distension and pain	38.5	13,300	Edema of colon, ileus/X-ray + CT
26. [16]	2009	34/M	Cystic fibrosis, DM	Abdominal distension, nausea with vomiting	39.1	15,200	Thickening of the wall of the entire colon/X-ray + CT
27. [17]	2010	82/F	-	Abdominal discomfort, nausea	Yes	50,000	-
28. [18]	2012	80/M	HT, stroke, prostate ca without dissemination	Abdominal distension, constipation	No	10,600	Colitis/CT
29. [19]	2016	64/F	COPD, HT, pancreatitis with colonic fistula and colostomy	Abdominal pain, nausea	No	30,000	Small bowel enteritis/CT
30. [20]	2018	66/M	Asthma, chronic pain, DM	Abdominal pain, nausea	No	13,000	Colonic wall dilatation/CT
31. [21]	2020 ⁑	-	Cancer, DM, liver cirrhosis (details unknown)	Ileus	-	-	-
32. [21]	2020 ⁑	-		Ileus	-	-	-
33. [21]	2020 ⁑	-		Ileus	-	-	-
34. [21]	2020 ⁑	-		Ileus	-	-	-
35. [21]	2020 ⁑	-		Ileus	-	-	-
36. [21]	2020 ⁑	-		Ileus	-	-	-
37. [22]	2021	75/F	HT, arthritis	Constipation	-	-	-
38. [22]	2021	73/M	HT, hyperlipidemia	Constipation	Yes	-	CT
39. [22]	2021	58/M	Liver cirrhosis	Constipation, abdominal distension	-	-	-
40. [22]	2021	49/M	None	Constipation, abdominal distension, acute renal failure	-	12,300	-
41. [22]	2021	62/M	Pituitary tumor	Obstipation, abdominal distension, acute renal failure	-	22,000	-
42. [22]	2021	79/M	HT, DM, hyperlipidemia	Constipation, abdominal distension	-	27,700	-
43. [22]	2021	77/F	Breast ca, hyperlipidemia	Abdominal distension, constipation	-	-	Colitis/CT
44. [22]	2021	81/F	Squamous cell carcinoma, HT	Abdominal distension, constipation	-	69,900	-
45. [22]	2021	78/F	Colon ca, chronic renal failure, HT	Abdominal distension and pain	-	31,200	-
46. [23]	2022	22/F	Dysmenorrhea	Abdominal distension, constipation	No	-	Small and large bowel dilatated/CT
47. [24]	2022	74/F	COPD, SLE, methadone addiction	Constipation, abdominal distension	No	17,900	Colitis
48. [25]	2024	23/F	Pregnancy	Abdominal distension, constipation	-	-	Right and transverse colon dilatated/ MR
49. [PRESENTED CASE]	2024	36/F	None	Abdominal distension and pain, constipation	38.4	14,100	Small and large bowel dilatation/CT

* In that study, there were 12 patients diagnosed with CDI and 5 of them presented without diarrhea. However, the patients’ individual properties were missing. ⁑ Lee et al. reported 6 CDI cases who presented with ileus; details were not mentioned case by case in the article. Missing data are indicated as “-”. Abbreviations: AML: acute myeloid leukemia; Ca: cancer; COPD: chronic obstructive pulmonary disease; CT: computerized tomography; DM: diabetes mellitus; F: female; HT: hypertension; M: male; NHL: non-Hodgkin lymphoma; Ref: reference; SLE: systemic lupus erythematosus; USG: ultrasonography; X-ray: direct graphy.

**Table 2:** Diagnosis, management, and outcomes of 48 patients diagnosed with CDI in the absence of diarrhea.

Case No [Ref]	Method of Diagnosis	Sampling of Stool	Inciting Antibiotic	Colonoscopy	Management	Outcome
[4]	Stool toxin + blood culture	-	Gentamicin–fluxocacillin–trimethoprim–sulfamethoxazole	Not performed	Vancomycin PO + metronidazole	Exitus
2. [4]	Stool toxin	-	Gentamisin–piperacillin	Not performed	Vancomycin PO + metronidazole IV	Complete recovery
3. [5]	Toxin * + colonoscopy	-	Piperacillin + vancomycin + ceftazidime	PMC	Vancomycin NG tube + metronidazole IV	Complete recovery
4. [5]	Toxin + colonoscopy	-	Trimethoprim–sulfamethoxazole	PMC	Vancomycin NG tube + metronidazole IV	Complete recovery
5. [5]	Toxin + colonoscopy	-	Trimethoprim–sulfamethoxazole	PMC absent, moderate mucosal erythema, edema	Metronidazole IV	Complete recovery
6. [5]	Colonic aspirate toxin + colonoscopy	-	-	PMC	Vancomycin NG tube + metronidazole IV	Complete recovery
7. [5]	Toxin + colonoscopy	-	Imipenem–cilastatin	PMC	Metronidazole IV	Complete recovery
8. [5]	Toxin + colonoscopy	-	Clindamycin	PMC	Metronidazole IV	Complete recovery
9. [6]	-	-	-	-	-	-
10. [6]	-	-	-	-	-	-
11. [6]	-	-	-	-	-	-
12. [6]	-	-	-	-	-	-
13. [6]	-	-	-	-	-	-
14. [7]	Postmortem histopathology	-	Cefotaxime	Not performed	Metronidazol IV	Exitus
15. [8]	Stool toxin + autopsy consistent with PMC	After laxatives	Erytromycin	Not performed	-	Exitus
16. [9]	∞ Pathology + stool culture	-	Not specified but + history of previous antibiotic use	-	Anticlostridial treatment for all (not specified)	Complete recovery
17. [9]	Stool culture	-	Not specified but + history of previous antibiotic use	Not performed	Anticlostridial treatment for all (not specified)	Complete recovery
18. [9]	Stool culture	-	Not specified but + history of previous antibiotic use	Not performed	Anticlostridial treatment for all (not specified)	Complete recovery
19. [10]	Stool toxin	Manually disimpacted	Levofloxacin	Not performed	Vancomycin NG and rectally + metronidazole IV	Exitus
20. [11]	Colonic biopsy toxin + colonoscopy	Rectal tube placement	Clindamycin + ceftizox	PMC	Metronidazole IV	Complete recovery
21. [12]	Stool and colonic biopsy toxin + colonoscopy	After therapeutic high-fiber diet	No previous antibiotic use	PMC	Vancomycin IV + metronidazole IV + subtotal colectomy	Complete recovery
22. [13]	Stool toxin + colonoscopy	During colonoscopy	Cefaperazon + amikacin + meropenem	PMC	Vancomycin intracolonic + metronidazole and vancomycin through nasoileus tube	Complete recovery
23. [14]	Surgical specimen pathology + for PMC	Manual evacuation and stool from stoma	Aztreonam + clindamycin	Not performed	Metronidazole, laparotomy and colectomy	Exitus
24. [14]	Stool toxin	-	Piperacillin–tazobactam, flucloxacillin	Not performed	Subtotal colectomy, metronidazole	Complete recovery
25. [15]	Stool toxin + proctoscopy	-	Cefazolin, amoxicillin–clavunate	PMC	Metronidazole IV, colectomy and temporary ileostomy	Complete recovery
26. [16]	Stool toxin + pathological examination of surgical specimen	Not mentioned	Trimethoprim–sulfametoxazole + ceftazidime	PMC	Metronidazole IV, subtotal colectomy	Exitus
27. [17]	Colonoscopy	Not performed	Vancomycin, ciprofloxacin	PMC	Metronidazole IV, total colectomy	Exitus
28. [18]	Stool culture + toxin + sigmoidoscopy	Stool from ileostomy	Cefuroxime, cephalexin	PMC	Total colectomy, permanent ileostomy, vancomycin and metronidazole	Recovered
29. [19]	Stool PCR	From colostomy	Trimethoprim–sulfamethoxazole + clindamycin+ vancomycin	Not performed	Vancomycin PO	Complete recovery
30. [20]	Stool PCR + sigmoidoscopy	During colonoscopy	-	PMC	Vancomycin PO + metronidazole IV	Complete recovery
31. [21] **	Stool PCR	-	-	-	-	Exitus
32. [21]	Stool PCR	-	-	-	-	Exitus
33. [21]	Stool PCR	-	-	-	-	Exitus
34. [21]	Stool PCR + Colon tissue PCR	-	-	-	Colectomy	Complete recovery
35. [21]	-	-	-	-	-	Complete recovery
36. [21]	-	-	-	-	-	Complete recovery
37. [22]	Stool toxin gene	Various ways ⁑	Vancomycin, levofloxacin, Aztreonam	-	Metronidazole	Complete recovery
38. [22]	Stool toxin gene	Various ways ⁑	Metronidazole, vancomycin, levofloxacin	-	Metronidazole	Complete recovery
39. [22]	Stool toxin gene	Loose stool after starting lactulose	Vancomycin, cephalexin	-	Metronidazole, recurrence, vancomycin PO + rectally	Recurrence, complete recovery
40. [22]	Stool toxin gene	Manual disimpaction	Linezolid, cefepime, levofloxacin	-	Metronidazole	Complete recovery
41. [22]	Stool toxin gene	Manual disimpaction	Cefazolin	-	Total colectomy + ileostomy	Complete recovery
42. [22]	Stool toxin gene	Stool from rigid rectal tube	Vancomycin, cefepime, levofloxacin	-	Total colectomy + ileostomy	Complete recovery
43. [22]	Stool toxin gene	Stool sample after enema	Vancomycin, cefepime, levofloxacin	-	Metronidazole, total colectomy	Exitus
44. [22]	Stool toxin gene	Stool sample after suppository	Cefazolin, metronidazole	-	Total colectomy	Exitus
45. [22]	Stool toxin gene	First stool from ileostomy after surgery	Cefazolin, metronidazole	-	Total colectomy	Exitus
46. [23]	Stool toxin	After colonoscopic decompression, watery bowel movement	Ciprofloxacin	PMCnot detected, nonspecific ileitis	Vancomycin PO	Complete recovery
47. [24]	Stool toxin and PCR	Single loose bloody stool	Ceftriaxone, Azithromycin	Not performed	Vancomycin PO	Complete recovery
48. [25]	Stool toxin and PCR	After a tap water enema	Ceftriaxone	Not performed	Vancomycin PO	Complete recovery
49. [PRESENTED CASE]	Stool toxin and colonoscopy	Rectal tube	Cefdinir	PMC	Vancomycin PO + metronidazole IV, surgical exploration	Complete recovery

* Method of diagnosiswas not specified in 5 of 6 cases, but it was stated that stool and colonic aspirates were collected from all patients. ** Three of six patients were treated with metronidazole IV; none of them was treated with vancomycin PO; treatment outcome was not specified per patient based on antimicrobial choice. ⁑ Various methods of stool collection included samples after suppository, enema, and manual disimpaction. ∞ Whether a biopsy was obtained or not was not specified. Abbreviations: IV: intravenous; PMC: pseudomembranous colitis; PO: per oral; Ref: reference. Missing data are indicated as “-”.

## Data Availability

Data will be shared on request from the authors.
